# The mediating role of social connectedness and negative cognitive emotion regulation in the association between problematic Internet use and depression among adolescents

**DOI:** 10.3389/fpubh.2024.1416073

**Published:** 2024-09-26

**Authors:** Jiaqi Xu, Xia-Can Chen, Lihua Chen, Dan Luo, Wenxin Bao, Xia Yang, Junzhe Ran, Jiajun Xu

**Affiliations:** ^1^Mental Health Center, West China Hospital, Sichuan University, Chengdu, China; ^2^West China School of Basic Medical Sciences and Forensic Medicine, Institute of Forensic Medicine, Sichuan University, Chengdu, China; ^3^Department of Neurology, Nantong Haimen People's Hospital, Nantong, China

**Keywords:** problematic Internet use, depression, adolescents, social connectedness, cognitive emotion regulation, mediation model

## Abstract

**Introduction:**

While the relationship between adolescent problematic Internet use (PIU) and depression has been extensively researched, few studies have investigated the role and mechanisms of social connectedness (SC) in this context. This study aimed to investigate the mediating effect of social connectedness (SC) and cognitive emotion regulation (CER) in the relationship between PIU and depression.

**Methods:**

We conducted a cross-sectional study involving 9,407 adolescents aged 12–18 years in China from September 2022 to March 2023. We employed Young’s 20-item Internet Addiction Test (IAT-20), the Social Connectedness Scale-Revised (SCS-R), the Cognitive Emotion Regulation Scale (CER), and the DSM-5 Level-2 Depression Scale to assess mental health outcomes. Logistic regression analysis was also performed to examine the independent association between the measured variables and depression. Mediation analysis was then conducted to evaluate the mediating roles of social connectedness and cognitive emotion regulation in the relationship between PIU and depression.

**Results:**

We found that the prevalence of PIU was 21.8%. Offline SC (indirect effect: 0.112, 95% CI: 0.104–0.121) and negative CER (indirect effect: 00.140, 95% CI: 0.129–0.152) mainly played a parallel mediating role in the relationship between PIU and depression, along with online SC (on_SC) (indirect effect: 0.007, 95% CI: 0.005–0.010).

**Discussion:**

These findings provide valuable insights into how PIU is associated with depression and highlight the importance of fostering real-life interpersonal interactions. However, the generalizability of this study’s findings to other populations may be limited due to cultural factors.

## 1 Introduction

Problematic Internet use (PIU), commonly identified as a new form of addictive behavior (1–3), is characterized by an individual’s inability to control their Internet use, which in turn leads to feelings of distress and functional impairment in daily activities (4). As of December 2023, China had 1,079 million netizens, of whom 13.9% were adolescents (5). With increasing access to online media, studies indicate a growing prevalence of PIU among adolescents in both Eastern and Western countries (6, 7). Moreover, rising PIU levels contribute to various psychosocial and mental health issues in adolescents (8). However, few studies have explored how PIU increases depression levels among adolescents. Previous surveys on adolescent PIU in China, limited to one or two provinces, may not represent the nation as a whole (9–11).

Individuals with PIU are more likely to develop depression (8, 12), with a recent longitudinal survey revealing that (12) adolescents diagnosed with depressive disorders showed greater susceptibility to PIU.

For instance, a study conducted in China involving 17,058 middle school students found that adolescents with Internet addiction were more likely to experience depression, with anxiety partially mediating this relationship (13). The complex mechanisms underlying the relationship between PIU and depression have garnered significant research attention. In addition, some studies have shown that a lack of social connection is associated with increased and prolonged Internet usage (14, 15). Social connectedness (SC), defined as an individual’s subjective perception of interpersonal relationships (16), is considered a protective factor against depressive symptoms (17, 18). The rise of online games and social media has driven adolescents to focus more on online interactions, often at the expense of face-to-face communication (19), a trend further intensified by the COVID-19 pandemic (20, 21). This shift raises questions about whether online social connectedness (on_SC) has the same positive effects as offline social connectedness (off_SC). To date, very little attention has been paid to the role of on_SC and off_SC in the relationship between PIU and depression among adolescents.

Higher levels of SC have been associated with the use of positive coping strategies when facing challenges (22, 23), whereas social isolation often indicates maladjustment (24).

Cognitive emotion regulation (CER) determines the coping strategies an individual uses to manage the intensity and nature of emotional experience (25–27). Previous evidence has identified that negative emotion regulation strategies are strongly associated with depression (26, 28). Moreover, individuals with PIU are more likely to adopt negative coping strategies, such as denial and behavioral disengagement (29, 30).

Several mediating factors in the association between PIU and depression have been identified, such as fear of missing out, sleep disturbances, anxiety, and bodily pain (13, 31–33). This raises the question of whether SC and CER could also serve as potential mediators in this relationship, particularly among adolescents who are undergoing significant psychological transitions and require focused attention. However, existing studies have not yet fully elucidated the mediating roles of both online and offline SC, as well as CER, in the connection between PIU and depression. Investigating the interplay between SC and CER may offer valuable insights for developing more effective prevention strategies.

Therefore, the primary objective of this study was to determine the prevalence of PIU among adolescents aged 12–18 years in China and to assess its impact on depression. The secondary objective was to examine the potential mediating effects of SC and CER, as well as to explore their interaction within the relationship between PIU and depression. Additionally, we aimed to investigate any differences between online and offline SC as mediators.

## 2 Methods

### 2.1 Study design and participants

**Figure 1:**
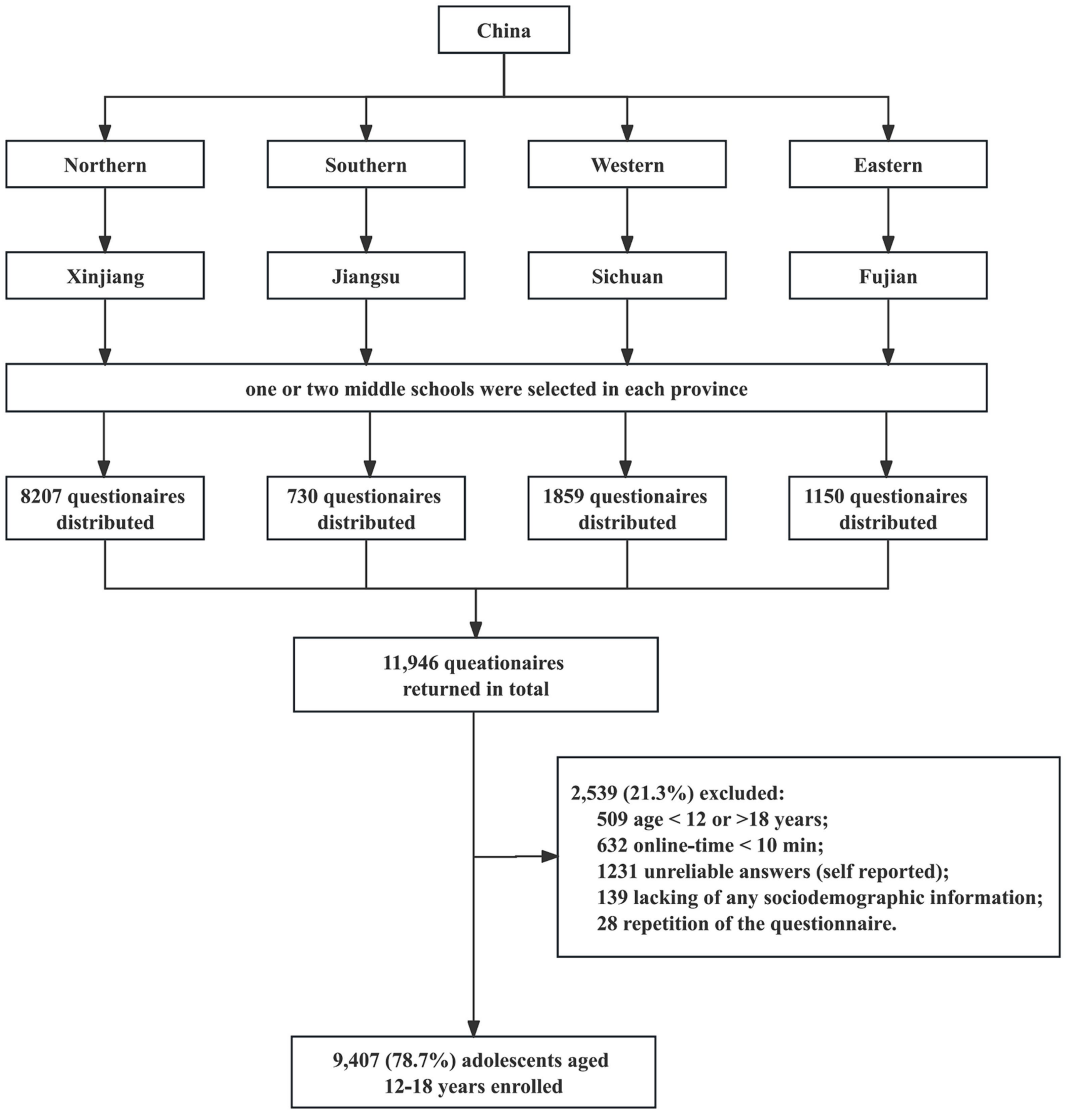
Flowchart of the procedure for data collection.

This cross-sectional study was conducted across four Chinese provinces—Fujian, Jiangsu, Sichuan, and Xinjiang—representing the eastern, southern, western, and northern regions of China, respectively, from September 2022 to March 2023. Ethical approval was obtained from the Ethics Committee of West China Hospital, Sichuan University (NO. 2019-907). In each province, one to two middle schools were selected for participation in the study (Figure 1). We distributed self-reported anonymous questionnaires to all students aged 12–18 years at each school. A designated computer class was arranged in each selected school, where students individually completed the online questionnaire through the Chinese web survey platform wjx.cn under their teacher’s supervision. All measures were administered in Mandarin Chinese. Both participants and their guardians provided informed consent before the survey.

A total of 11,946 adolescents were included in this survey, which was administered online using non-skippable questions to ensure no missing responses. Based on the following criteria, 2,539 questionnaires were excluded: (1) participants reporting an age younger than 12 or older than 18; (2) participants who completed the survey in less than 10 min; (3) participants who indicated that their responses were “false” or “entirely false” when asked, “To what extent does your survey reflect the real situation?” (entirely false, false, general, true, entirely true) at the end of the survey; (4) and questionnaires missing key demographic information. Additionally, questionnaires with matching attributes—such as name, age, sex, height, weight, and school affiliation—were considered duplicates, and only the most complete data set was retained for further analysis. Ultimately, data from 9,407 participants (14.90 ± 1.61 years) were included in this study, representing 78.7% of the total sample.

### 2.2 Measurements

#### 2.2.1 Sociodemographic variables

A self-administered questionnaire was used to collect sociodemographic variables, including age, sex, only-child status, handedness, place of residence, father’s education level, and mother’s education level.

#### 2.2.2 Young’s 20-item Internet Addiction Test

The IAT-20, developed by Oliveira et al. (34), is a globally recognized self-report tool for assessing PIU or Internet addiction. It consists of 20 questions, each scored on a scale from 1 to 5 (1 = rarely, 2 = occasionally, 3 = frequently, 4 = often, 5 = always). The tool demonstrated strong internal consistency with a Cronbach’s *α* coefficient of 0.90 (35). Based on previous studies, a total score of ≥50 indicates moderate to severe Internet dependence, which was classified as PIU in this study (36, 37).

#### 2.2.3 Social Connectedness Scale—Revised

The Social Connectedness Scale Revised (SCS-R), developed by Lee and Robbins (38), is a widely used tool for measuring SC. It contains 20 items rated on a 6-point scale, ranging from 1 (strongly disagree) to 6 (strongly agree), with higher scores indicating greater SC. The scale was translated into Chinese and validated, demonstrating a Cronbach’s *α* of 0.92 (39). We measured on_SC and off_SC by prefixing each question with “in the online world” or “in the real world,” respectively. The social connectedness gap (SC_gap) was calculated as the difference between the on_SC and off_SC scores to reflect the individual’s SC tendency.

#### 2.2.4 Cognitive Emotion Regulation Questionnaires

The Cognitive Emotion Regulation Questionnaire (CERQ) was used to evaluate CER strategies applied in response to stressful events (26). The CERQ contains nine subscales, categorized into negative and positive (25) types of regulation (nCER and pCER), with each time rated on a 5-point Likert scale. The Chinese version of the CERQ has been validated, demonstrating a Cronbach’s *α* of 0.89 (40).

#### 2.2.5 DSM-5-TR Level-2 Depression Scale

The DSM-5-TR Level 2—Depression—Child Age 11–17 measure is a 14-item patient-reported outcome measurement tool from the PROMIS Depression Short Form, developed by the American Psychiatric Association (APA), designed to assess depression in children and adolescents (41). The scale is intended for children aged 8–17 years, with each item rated on a 5-point scale (1 = never; 2 = almost never; 3 = sometimes; 4 = often; and 5 = almost always). Higher scores indicate greater severity of depression. The Chinese version was translated and validated by Wang and Zhong (42). Participants with T-scores above 55 were classified as having mild to severe depression (41).

### 2.3 Data analysis

Categorical variables were described using numbers (percentages), while continuous variables were presented as means (standard deviations). Social connectedness (SC) measurements included on_SC, off_SC, and SC_gap (calculated as the difference between the on_SC and off_SC scores). Based on whether the SC_gap score was positive or negative, participants were categorized into the “more online” and “more offline” groups, respectively. Univariate binary logistic regression analysis was performed to identify whether PIU, social connectedness, pCER and nCER were associated with depression among adolescents. Then, the multivariate logistic regression analysis was conducted to identify whether PIU, social connectedness, pCER and nCER were independently associated with depression among adolescents. Spearman’s correlation was conducted to examine mutual correlations between sociodemographic variables, PIU, SC measures, CER, and depression.

Variables that were significantly associated with depression in the multivariate logistic regression were subsequently included in the structural equation modeling (SEM) to examine the mediating effects of SC and CER in the relationship between PIU and depression. The effect size in the mediation analysis was assessed using the STDYX standardized effect size.

The fit of the hypothesis models was evaluated using the chi-squared degrees of freedom ratio (χ^2^/df), the Comparative Fitting Index (CFI), the Tacker-Lewis’s Index (TLI), the Root Mean Square Error of Approximation (RMSEA), and the Standardized Root Mean Square Residual (SRMR). In this study, satisfactory goodness of fit was indicated by χ^2^/df ≤ 3, CFI ≥ 0.95, TLI ≥ 0.95, RMSEA≤0.05, and SRMR ≤ 0.05.

A total of 2,000 bootstraps were conducted, and 95% confidence intervals (95% CI) of the estimates were calculated. Sociodemographic variables that were independently associated with depression in multivariate regression were adjusted for in all SEM models.

Group differences, regression, and correlation analysis were conducted using SPSS 26.0, while SEM analysis was conducted using Mplus 8.0 (43). All tests were two-tailed, with the significant threshold set at *α* = 0.05.

## 3 Results

### 3.1 Demographic characteristics

The prevalence of depression (mild to severe) and PIU were 35.8 and 21.8%, respectively. Table 1 shows the participants’ sociodemographic characteristics, SC, pCER, nCER, and PIU.

**Table 1:** Characteristics of adolescents aged 12–18 years and the association between depression and other variables.

Variables	Overall (*n* = 9,407)	Depression (*n* = 3,372)	ORs^a^ (95% CI)	AORs (95% CI)
Age	14.90 ± 1.61	15.06 ± 1.55	1.10 (1.07–1.13)^***^	1.01 (0.98–1.04)^b^
**Sex**				
Male	4,556 (48.4%)	1,371 (40.7%)	Ref.	Ref.
Female	4,851 (51.6%)	2,001 (59.3%)	1.63 (1.50–1.78)^***^	1.96 (1.77–2.17)^b^^,***^
**Handedness**				
Right	8,941 (95.0%)	3,178 (94.2%)	Ref.	Ref.
Left	466 (5.0%)	194 (5.8%)	1.29 (1.07–1.56)^*^	1.48 (1.16–1.89)^b^^,**^
**Only-child**				
No	5,173 (55.0%)	1905 (56.5%)	Ref.	Ref.
Yes	4,234 (45.0%)	1,467 (43.5%)	0.91 (0.84–0.99)^*^	0.96 (0.86–1.08)^b^
**Residence**				
Urban	7,429 (79.0%)	2,504 (74.3%)	Ref.	Ref.
Town	1,264 (13.4%)	492 (14.6%)	1.25 (1.11–1.41)^***^	0.99 (0.85–1.16)^b^
Rural	714 (7.6%)	376 (11.2%)	2.22 (1.88–2.55)^***^	1.20 (0.99–1.47)^b^
**Father’s education level**				
0–9 years	3,379 (35.9%)	1,376 (40.8%)	Ref.	Ref.
10–12 years	2,390 (25.4%)	852 (25.3%)	0.81 (0.72–0.90)^***^	1.03 (0.89–1.20)^b^
13–17 years	3,481 (37.0%)	1,086 (32.2%)	0.66 (0.60–0.73)^***^	0.88 (0.74–1.04)^b^
Over 17 years	157 (1.7%)	58 (1.7%)	0.85 (0.61–1.19)	0.79 (0.51–1.22)^b^
**Mother’s education level**				
0–9 years	3,650 (38.8%)	1,492 (44.2%)	Ref.	Ref.
10–12 years	2,126 (22.6%)	727 (21.6%)	0.75 (0.67–0.84)^***^	0.93 (0.80–1.09)^b^
13–17 years	3,545 (37.7%)	1,120 (33.2%)	0.67 (0.61–0.74)^***^	0.89 (0.75–1.05)^b^
Over 17 years	86 (0.9%)	33 (1.0%)	0.90 (0.58–1.40)	1.28 (0.71–2.31)^b^
on_SC	77.65 ± 11.91	75.50 ± 11.45	0.61 (0.57–0.66) ^***^	0.71 (0.65–0.78)^c^^,***^
off_SC	84.92 ± 16.10	75.75 ± 14.54	0.25 (0.24–0.27)^***^	0.34 (0.31–0.37)^b^^,***^
**SC_gap**				
More online	3,306 (35.1%)	1746 (51.8%)	3.08 (2.82–3.37)^***^	2.22 (2.02–2.41)^d^^,***^
More offline	6,101 (64.9%)	1,626 (48.2%)	Ref.	Ref.
nCER	41.52 ± 11.17	48.30 ± 8.76	7.35 (6.63–8.16)^***^	4.96 (4.34–5.66)^b^^,***^
pCER	63.44 ± 13.80	66.02 ± 11.12	1.57 (1.47–1.68)^***^	0.92 (0.82–1.04)^b^
**PIU**				
No	7,352 (78.2%)	2,112 (62.6%)	Ref.	Ref.
Yes	2,055 (21.8%)	1,260 (37.4%)	3.93 (3.55–4.36)^***^	1.64 (1.51–1.79)^b^^,***^

on_SC, online social connectedness; off_SC, offline social connectedness; SC_gap, online-offline social connectedness gap (on_SC score minus the off_SC score); nCER, negative cognitive emotion regulation; PIU, problematic Internet use, the total score of Internet addiction test (IAT-20); OR, odds ratio; CI, confidence interval.

a OR (95% CI) based on univariate binary logistic regression using depression-or-not as a dependent variable.

b Adjusted ORs (95% CI) based on multivariable logistic regression using depression-or-not as a dependent variable, sociodemographic factors, off_SC, nCER, pCER, and PIU.

c Adjusted ORs (95% CI) based on multivariable logistic regression using depression-or-not as a dependent variable, sociodemographic factors, on_SC, nCER, pCER, and PIU.

d Adjusted ORs (95% CI) based on multivariable logistic regression using depression-or-not as a dependent variable, sociodemographic factors, SC_gap, pCER, nCER, and PIU.

^*^p < 0.05, ^**^p < 0.01, and ^***^p < 0.001.

### 3.2 Associated factors of depression

According to univariate logistic regression, on_SC, off_SC, SC_gap, nCER, pCER, and PIU showed significant associations with depression (*p* < 0.05) (Table 1).

After adjusting for confounding factors (sex, handedness, and residence), SC_gap, nCER, and PIU had independently and significantly positive associations with depression (*p* < 0.001), while both on_SC and off_SC had independently and significantly negative associations with depression among adolescents (*p* < 0.001) (Table 1). Adolescents with higher SC_gap scores were associated with depression after controlling the confounding factors (AOR:2.45, 95% CI:2.25–2.67, *p* < 0.001). Notably, pCER did not significantly correlate with depression in multivariable logistic regression.

### 3.3 Correlation analysis

The results of Spearman’s correlation analysis are shown in Table 2. Both on_SC and off_SC exhibited a negative correlation with PIU and depression, while the SC_gap demonstrated a significant positive correlation with both PIU and depression (*p* < 0.001). Additionally, older age, greater distance from urban areas, and lower levels of parental education were found to be significantly associated with higher levels of PIU and depression.

**Table 2:** Spearman’s correlation among sociodemographic variables, PIU, depression, nCER, and SCs.

	Age	Sex	Handedness	Only-child	Residence	Father’s education level	Mother’s education level	on_SC	off_SC	SC_gap	nCER	PIU
on_SC	−0.03^**^	−0.03^**^	0.03^**^	−0.00	−0.04^***^	0.01	0.01	-				
off_SC	−0.09^***^	−0.02^*^	0.02	0.03^***^	−0.11^***^	0.10^***^	0.10^***^	0.42^***^	-			
SC_gap	0.07^***^	−0.01	−0.01	−0.04^***^	0.09^***^	−0.10^***^	−0.10^***^	0.23^***^	−0.72^***^	-		
nCER	0.10^***^	0.02	0.01	0.01	0.09^***^	−0.05^***^	−0.06^***^	−0.06^***^	−0.32^***^	0.29^***^	-	
PIU	0.17^***^	−0.03^*^	0.00	0.05^***^	0.09^***^	−0.02^*^	−0.03^**^	−0.05^***^	−0.35^***^	0.32^***^	0.44^***^	-
Depression	0.09^***^	0.13^***^	−0.02	−0.00	0.10^***^	−0.07^***^	−0.08^***^	−0.11^***^	−0.42^***^	0.35^***^	0.55^***^	0.42^***^

on_SC, online social connectedness; off_SC, offline social connectedness; SC_gap, online-offline social connectedness gap; nCER, negative cognitive emotion regulation; PIU, problematic Internet use, the total score of the Internet Addiction Test (IAT-20).

^*^p < 0.05, ^**^p < 0.01, ^***^p < 0.001.

### 3.4 Structural equation modeling

Mediation analysis showed that three chain mediation models were all verified as satisfactory goodness of fit. PIU was directly associated with depression in Models A, B, and C (*β* = 0.212, 0.130, and 0.161, respectively, *p* < 0.001) after adjusting for sex, handedness, and residence.

**Figure 2:**
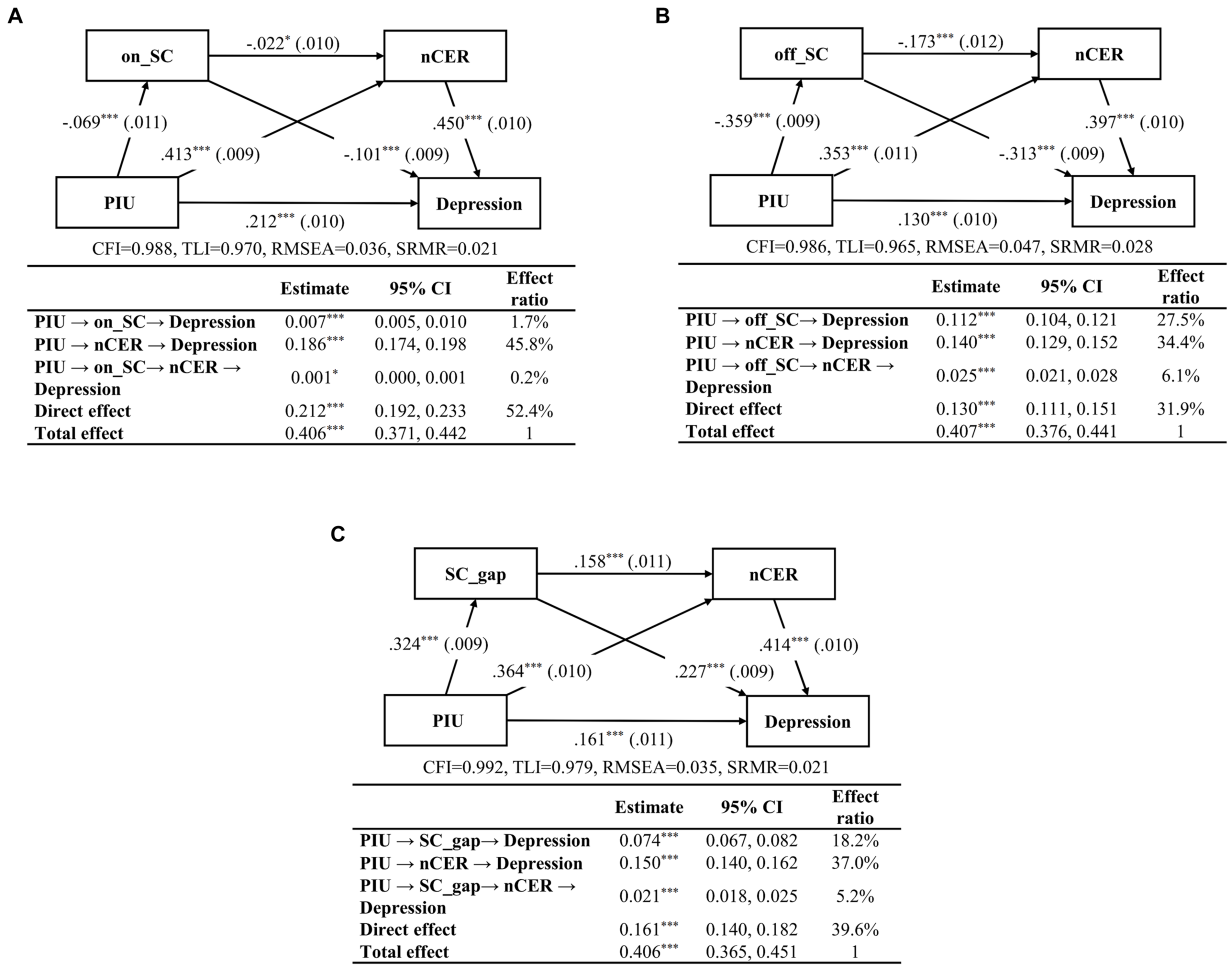
The mediation effects of SC (**A**, on_SC; **B**, off_SC; **C**, SC_gap) and nCER on the relationship between PIU and depression in adolescents aged 12–18 years. The statistical significance of the mediation was adjusted for sex, handedness, and residence, with standardized coefficients (*β*), and standard error (S.E.) presented in parentheses. The degree of fitting along with indirect and direct effects are displayed under each model. PIU, problematic Internet use, the total score of the Internet Addiction Test (IAT-20); nCER, negative cognitive emotion regulation; on_SC, online social connectedness; off_SC, offline social connectedness; SC_gap, online-offline social connectedness gap. CI, confidence interval. ^*^p < 0.05, ^***^p < 0.001.

In Model A, both on_SC and nCER significantly mediated the relationship between PIU and depression (Figure 2A). Notably, the single mediating pathway of nCER independently contributed to 45.8% of the overall effect, whereas on_SC alone contributed 1.7%. In contrast, the chained mediation pathway (PIU on_SC-nCER-depression) accounted for a minor proportion of the total effect (0.2%).

In Model B, off_SC and nCER were identified as mediators (Figure 2B). The single mediating effect of nCER accounted for 34.4% of the total effect, closely followed by the single mediation effect of off_SC (27.5%). Their chained mediation model (PIU-off_SC-nCER-depression) was only 6.1%, which was much smaller than the single mediating pathway.

In Model C, the single mediating effect of nCER accounted for 37.0% of the total effect (Figure 2C). The SC_gap significantly mediated the connection between PIU and depression, accounting for 18.2% of the total effect. The chained mediating effect from the SC_gap to nCER was lower at 5.2%, compared to their individual effects.

## 4 Discussion

This study, based on a national sample of 9,407 adolescents in China, yielded novel findings indicating that off_SC, SC_gap, and nCER mainly played parallel mediating roles in the relationship between PIU and depression. Although on_SC also had a significant mediating effect, its role was less prominent compared to off_SC. These findings provide a new perspective on how PIU contributes to depression, highlighting the critical importance of fostering real-life interpersonal interactions to mitigate its negative impact on adolescents.

According to this study, the prevalence of PIU among adolescents was 21.8% (95% CI: 20.9–22.6%). This is consistent with a recent survey involving 3,357 Chinese adolescents, which reported a PIU rate of 26.09% (44). However, this rate is higher than other surveys conducted among Chinese adolescents, such as 6.0% in a sample of 755 adolescent Internet users from Wuhan (9) and 6.3% in a study involving 2,758 adolescents from southern China (11).

These discrepancies may partly result from variations in the sampling methods and instruments used. The previous study employed the same questionnaire and PIU cut-off score as ours. However, their sample size was relatively small. In contrast, the latter study employed a parental assessment questionnaire (45), which could introduce bias compared to self-reported data. Our study utilized the IAT-20, a widely validated instrument across countries (46–48), and was conducted in diverse regions of China rather than being confined to a single city or district. Cultural factors, such as the limited exposure of Chinese middle school students to electronics on weekdays, which may reduce PIU risk, should also be considered.

A study conducted in Spain showed a higher PIU rate of 33% among adolescents (49). Similar to findings from previous research, we found that older and male adolescents were more likely to experience PIU (6, 50–52), while girls were more likely to develop depression. Additionally, residing in a town or rural area was found to be associated with a higher prevalence of PIU compared to living in urban areas, which aligns with findings on the influence of residence on depression. Consistent with our results, a study using data from the National Children’s Study of China found that, among Internet users, male individuals and rural students reported higher rates of Internet addiction than female individuals and urban students (51).

To the best of our knowledge, this is the first study to confirm the mediating roles of both on_SC and off_SC and their gap in the relationship between PIU and depression. Our findings suggest that adolescents with PIU experienced a significant decline in off_SC, which is consistent with previous research indicating that individuals with PIU make minimal effort to maintain interpersonal relationships (19) and may face challenges related to social anxiety (53). Strong SC has been shown to yield positive effects, such as reducing the risk of suicide, enhancing happiness and life satisfaction, decreasing feelings of loneliness, and mitigating depressive symptoms (54–56). In line with previous literature, off_SC played a protective role in Model B of our study. Notably, on_SC also exhibited a weak negative association with both PIU and depression. Recent studies have similarly reported that on_SC plays a protective role in preventing depression, particularly among older adults (57).

Additionally, online social connections served as a buffer against depression and anxiety among young adults during the COVID-19 pandemic (58). However, the impact of online social networking remains controversial and appears to vary depending on its purpose (59, 60). Our study provided evidence that on_SC indeed had a weak protective effect in mitigating the impact of PIU on depression among adolescents. In contrast, the SC_gap, which reflects the disparity between perceived social closeness online and in real life, positively mediated the relationship between PIU and depression.

This novel finding indicates that PIU leads adolescents to prioritize online interactions over offline communication, thereby increasing the risk of depression. In line with our findings, previous studies have highlighted that face-to-face communication fosters high-quality listening, behavioral synchrony, and self-disclosure, which serve as protective factors against negative emotions (61). These findings suggest that even with extensive Internet usage, encouraging real-life social interactions could help mitigate the risk of depression among adolescents.

In addition, our study also demonstrated the mediating role of nCER in the relationship between PIU and depression. Previous research has linked nCER to PIU, including Internet gaming disorder (62–65). A literature review confirmed this association, suggesting that PIU may serve as a coping mechanism to compensate for emotional regulation deficits, thereby increasing the risk of nCER (66), a factor that has been understudied among adolescents.

Furthermore, those who rely on maladaptive strategies may be more susceptible to psychopathology (25, 26), which is particularly important for adolescents due to the developmental changes in their affective systems (67). As widely documented, chronic difficulties with CER during adolescence and late childhood are associated with depression (68–70). Our findings have confirmed the mediating role of nCER in the relationship between PIU and depression, suggesting that cognitive therapy aimed at reducing nCER could benefit adolescents with PIU and potentially prevent the onset of depression.

The present study also identified a weak chain mediating effect from SCs to nCER. A study involving 1,291 adolescents in Ireland found that adolescents with PIU experienced less perceived social support and greater difficulty in emotion regulation (71). However, the relationship between these two factors was not further examined. Neuroimaging studies have shown that increased social interactions among adolescents enhance emotional regulation capabilities, promote positive coping strategies (22) and reduce negative emotions (72).

Our SEM analysis supports these findings, demonstrating that off_SC is more effective than on_SC in reducing negative coping strategies, such as catastrophizing. Therefore, interventions aimed at enhancing off_SC should be developed for middle school students, especially those with PIU. For instance, engaging in dual sports activities has shown significant potential in reducing Internet addiction among adolescents (73).

## 5 Limitations

There are also some limitations to this study. First, the cross-sectional design makes it difficult to establish causality, and future research could benefit from longitudinal studies. Furthermore, adolescents with PIU may use the Internet as a platform for developing other addictions, such as gaming, online shopping, or watching short videos. Moreover, due to research resource constraints, we utilized non-random sampling methods in this study, which may introduce biases associated with specific school-related factors.

For instance, the educational quality and management approaches of these schools could influence students’ Internet usage and mental health. Disparities in the allocation of educational resources across provinces may also affect the generalizability of our findings. This study primarily focused on overall problematic Internet usage. Therefore, future studies should consider examining various forms of PIU (e.g., Internet gaming disorder and social media disorder) to gain a more comprehensive understanding of the effect of adolescent Internet-related behavioral issues on mental health.

## 6 Conclusion

Despite these limitations, our study is the first to investigate the potential mediating role of SC and CER strategies. We uncovered an important mechanism that helps explain why adolescents with PIU are more likely to develop depressive symptoms. Targeted interventions and strategies in clinical practice, focusing on enhancing adolescents off_SC and promoting the adoption of positive coping strategies in response to stressors, could help improve the overall mental wellbeing of adolescents.

## Data Availability

The raw data supporting the conclusions of this article will be made available by the authors, without undue reservation.
